# Long-term survival after treatment of unruptured intracranial aneurysms: A population-based perspective

**DOI:** 10.1016/j.bas.2026.106096

**Published:** 2026-05-19

**Authors:** Basil E. Grüter, Benedikt Bandhauer, Stefan Wanderer, Philipp Gruber, Jatta Berberat, Cordula Blohm, Marco Cattaneo, Gerrit A. Schubert, Luca Regli, Luca Remonda, Serge Marbacher, Lukas Andereggen, Hans-Jakob Steiger

**Affiliations:** a Division of Neuroradiology, Department of Radiology, Cantonal Hospital Aarau, Aarau, Switzerland; b Department of Neurosurgery, Cantonal Hospital Aarau, Aarau, Switzerland; c Federal Statistical Office, Neuchâtel, Switzerland; d Department of Clinical Research, University of Basel, University Hospital Basel, Basel, Switzerland; e Department of Neurosurgery, RWTH Aachen University, Aachen, Germany; f Department of Neurosurgery, Clinical Neuroscience Center, University Hospital Zurich, University of Zurich, Switzerland; g Division of Neuroradiology, Department of Radiology, Cantonal Hospital Aarau, University of Bern, Switzerland; h Department of Neurosurgery, Cantonal Hospital Aarau, University of Bern, Aarau, Switzerland; i Department of Neurosurgery, Heinrich-Heine-University, Düsseldorf, Germany

**Keywords:** Unruptured intracranial aneurysm, Mortality, Life expectancy, Cardiovascular death, Survival

## Abstract

**Introduction:**

Unruptured intracranial aneurysms (UIAs) affect 2–3% of the general population. While treatment decisions focus on balancing procedural risks with the long-term rupture risk, little is known about the overall life expectancy and long-term mortality in this patient group.

**Research question:**

This study aimed to evaluate survival, causes of death, and cardiovascular risk factors in UIA patients compared to the general population.

**Material and methods:**

In this observational study, 382 patients with UIAs were identified from a single-center cohort of 1107 treated intracranial aneurysms (2006–2022). Patients were matched 1:100 by age and sex to individuals from the general population. Survival and cause-of-death data were obtained from national records, with WHO-based classification. Cardiovascular risk factors were extracted from hospital records and compared to national health survey data.

**Results:**

Patients with UIAs (median age 59, 36% male) had a significantly higher mortality risk than the matched population (hazard ratio 3.116; CI 2.291-4.239; p < 0.001). Median life expectancy was 73.9 years [69.9, 79.1], 13 years shorter than the matched cohort (87.0 years [86.4, 87.4]). Cardiovascular disease was a more frequent cause of death in UIA patients (34.3% vs. 21.3%), and cardiovascular risk factors were highly prevalent.

**Discussion and conclusions:**

Patients with UIAs experience markedly reduced life expectancy and excess cardiovascular mortality. These findings emphasize the importance of integrating aggressive cardiovascular risk management into UIA care and call for cautious selection of preventive treatment, particularly in patients with significant comorbidities.

## Highlights

• Patients with UIAs show markedly reduced life expectancy versus the general population.
• Cardiovascular disease is the leading cause of excess mortality in UIA patients.
• Periprocedural mortality is low, but long-term vascular risk remains high.
• Aggressive cardiovascular risk management is essential in UIA care.

## 1 Introduction

Unruptured intracranial aneurysms (UIAs) are detected in approximately 2–3% of the general population (Vlak et al., 2011; Korja et al., 2014a). With the increasing use and sensitivity of modern neuroimaging, UIAs are being identified with growing frequency, often incidentally. The management of these lesions requires careful, individualized decision-making, in which the potential benefits of preventive intervention are balanced against the natural history of the aneurysm and the procedural risks associated with treatment.

The estimated risk of aneurysm rupture over a patient's lifetime depends on both the annual rupture risk and the individual's remaining life expectancy. Annual rupture rates are influenced by aneurysm-specific characteristics—such as size, location, and morphology—as well as by patient-related factors including smoking and arterial hypertension (Rinkel et al., 1998; Korja et al., 2014b; Ishibashi et al., 2009; Morita et al., 2012; Wiebers et al., 2003). For most non-giant aneurysms, reported annual rupture rates range widely, reflecting the heterogeneity of patient populations and aneurysm features studied. An accurate estimation of remaining life expectancy is therefore an important component of risk stratification, yet remains challenging in routine clinical practice.

Population-based life expectancy data are commonly used to inform treatment decisions, although their applicability to patients with UIAs has not been fully clarified. UIAs represent a form of cerebrovascular pathology and may coexist with other vascular risk factors, potentially influencing long-term outcomes (Pyysalo et al., 2013). While excess mortality following aneurysmal subarachnoid hemorrhage has been well documented (Pyysalo et al., 2013; Asikainen et al., 2023; Nieuwkamp et al., 2011), including in patients who achieve favorable neurological recovery, (Huhtakangas et al., 2015; Fernandez-Perez et al., 2022; Nieuwkamp et al., 2014), considerably less is known about long-term survival and causes of death in patients with UIAs who undergo elective treatment.

To address this knowledge gap, the present observational study investigates life expectancy in patients treated for UIAs and compares their survival with that of an age- and sex-matched cohort from the general population. In addition, causes of death and the prevalence of cardiovascular risk factors are analyzed to provide a broader perspective on long-term outcomes in this patient group. A better understanding of these factors may support more nuanced counseling and shared decision-making in the management of UIAs.

## 2 Material and methods

The dataset consists of 382 consecutive UIAs treated at our centre from January 2006 to May 2022. All participants were aged 18 or older. The study received approval from the institutional review board (Neurovascular Research Group, NeuroReserach Office NRO, Kantonsspital Aarau) and the Swiss Ethics Commission (EKNZ, Nr. 2020-02249), in accordance with the Declaration of Helsinki. Individual consent was waived due to the retrospective nature of the study and the primary analysis of data from patients who were deceased at the time of the study.

### 2.1 Patient and aneurysm data collection

Patient information, including demographic details (age, sex), cardiovascular risk factors (e.g., smoking, hypertension, hypercholesterolemia, diabetes, obesity), comorbidities (such as coronary heart disease, cerebrovascular disease, or peripheral artery disease), medications, and UIA-specific characteristics (size, location, morphology, calcification), as well as treatment method (open surgery endovascular, conservative therapy), was systematically extracted from electronic hospital and imaging records.

### 2.2 Post-treatment survival and mortality analysis

Patient survival and follow-up duration were assessed from the treatment date to the final follow-up. Survival was confirmed if any care interaction occurred within the hospital network within the past year. For cases without such data, verification was conducted through the Federal Statistical Office using the patient's social security number, with all linked variables pseudonymized. For deaths not recorded in the hospital system (e.g., those occurring outside the hospital or in a different institution), the Federal Statistical Office provided cause-of-death data categorized according to the World Health Organization's (WHO) top 10 causes of death in high-income countries. (WHO)

### 2.3 General population comparison and matching

Pseudonymized data from the Federal Statistical Office, including date of birth, sex, survival status, causes of death, and comorbidities, was used to establish a comparison cohort from the regional population (614,500–698,400 individuals, 2010–2020). UIA patients were matched 1:100 to the general population by birth year and sex, excluding individuals who moved into or out of the region during the observation period. Patients diagnosed outside this range or without sufficient follow-up were excluded. Matching ensured that each UIA patient could be paired with up to 3634 individuals, with a minimum of 727 and a maximum of 4996. The distribution of cardiovascular risk factors and comorbidities in the general population was taken from the results of the Swiss Health Survey 2022 for the age group of 55-64 year-olds (Schweizerische Gesundheitsbefragung, 2022).

### 2.4 Statistical analyses

Survival outcomes of UIA patients were compared to the matched population using Cox regression, accounting for calendar time. Missing follow-up data were treated as censored at the last recorded observation. The proportional hazards assumption for Cox models was validated using scaled Schoenfeld residuals. Causes of death in UIA patients were analyzed in relation to the matched population. For the survival analysis, the matching population remained fixed, but only the common follow-up time of patients and matches was considered.Frequency distributions for categorical variables were assessed with Pearson's chi-squared test, and numerical variables were analyzed with the Wilcoxon–Mann–Whitney test. Chi-Square Test for proportions was used to compare the prevalence of risk factors between populations. A non-standard test was used to test the difference in medians. (Chen and Zhang, 2016). The reported life expectancy calculations report medians. Analyses were performed using R (Team, 2022) software, applying two-sided tests with a significance level of 5% and 95% confidence intervals, without adjustments for multiple comparisons.

## 3 Results

This study included 382 patients that presented with 556 UIAs. They had a median age of 59 years [IQR 51, 66 years] and 36% were men. The comparison cohort from the general population was matched by birth year (and sex), and accordingly has exactly the same age and sex distribution. Demographic characteristics are shown in Table 1.

**Table 1:** Patient demographics and aneurysm characteristics Categorical data are given as absolute frequencies (%), numerical variables as median [interquartile range]. Patient data is from the time of first IA diagnosis, data for IA represents all diagnoses.

**Patient demographics**		
n		382
Age (years)		59.00 [51.00, 66.00]
Male sex (%?)		137 (35.9)
Death (%?)		51 (13.4)

**Aneurysm characteristics**		
n		556
Treatment modality		
	endovascular	241 (43.4)
	microsurgical	263 (47.3)
	conservative	52 (9.3)
Shape		
	saccular	469 (84.4)
	fusiform	86 (15.5)
	blister	1 (0.2)
Calcification (%?)		13 (2.3)
Partial thrombosed		27 (4.9)
Multilobarity IA (%?)		146 (26.3)
Location		
	Internal carotid artery (%)	142 (25.5)
	Middle cerebral artery (%)	176 (31.7)
	Anterior cerebral artery (%)	36 (6.5)
	Posterior communicating artery (%)	32 (5.8)
	Superior cerebellar artery (%)	2 (0.4)
	Vertebral artery (%)	9 (1.6)
	Basilar artery (%)	52 (9.4)
	Posterior inferior cerebral artery (%)	7 (1.3)
	Pericallosal artery (%)	19 (3.4)
	Anterior communicating artery (%)	80 (14.4)
	Posterior cerebral artery (%)	1 (0.2)
Maximal diameter IA in mm		6.00 [4.90, 8.45]

### 3.1 Survival of UIA patients

**Fig. 1:**
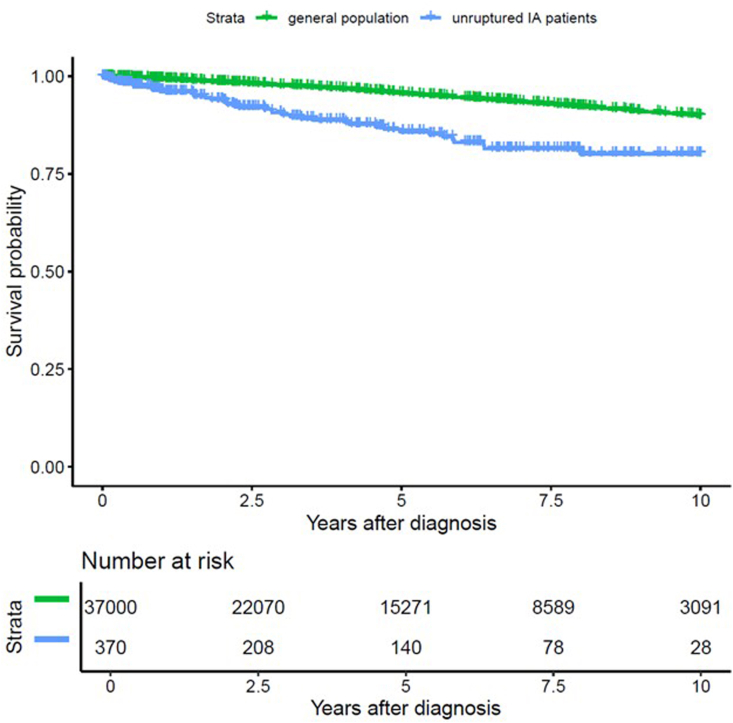
Kaplan–Meier curves illustrating the remaining life expectancy at the time of first diagnosis in patients with UIAs (blue), compared to the matched general population cohort (green). Patients with UIAs exhibit a significantly higher mortality rate, with survival markedly lower than that of the general population (p < 0.001).

**Fig. 2:**
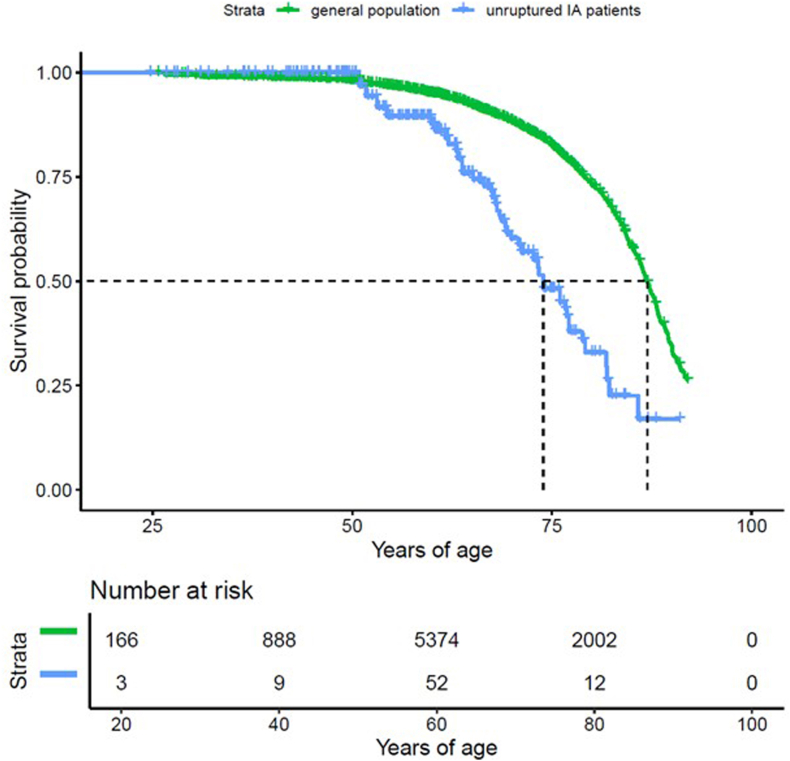
Life expectancy of IA patients compared to the matched cohort of the general population**.** Kaplan–Meier curves showing overall mortality for all patients. Dashed lines represent the median age at death with 95% confidence intervals: 73.9 years [69.9, 79.1] for patients UIAs (blue), and 87.0 years [86.4, 87.4]. for the matched general population (green).

From the 382 patients presenting with UIAs n = 9 were lost during follow-up (n = 5 moved abroad after UIA treatment and in n = 4 cases the social security number was missing or erroneous). Over the study period, the probability of survival in IA patients was significantly lower than in the matched general population (p < 0.001). Cumulative mortality rates were 22.9% for UIA patients and 8.4% for the matched population. (Fig. 1 and Supplementary Fig. 1). In comparison to the matched population, the hazard ratio of death (stratified by matching group) upon first diagnosis of an UIA was calculated to be 3.116 [CI 2.291, 4.239] (p < 0.001). The 30-day mortality after UIA treatment was 0.3% [0.0%, 0.8%] (n = 1). Procedure-related mortality was therefore exceptionally low in this contemporary cohort. While detailed long-term functional outcome and morbidity data were not consistently available for all retrospectively included patients, no evidence suggested that procedural complications represented a major contributor to the observed excess long-term mortality at the population level.The median age at death of patients with UIAs was 73.9 [69.9, 79.1] years as compared to 87.0 [86.4, 87.4].] years for the matched cohort of the general population (Fig. 2). While median life expectancy remains consistently reduced in treated UIA patients across all age thresholds, the relative survival gap narrows with increasing attained age (Table 2).

**Table 2:** Conditional median life expectancy of patients with treated UIAs compared with the age- and sex-matched general population at increasing index ages Median remaining life expectancy estimates are shown for individuals who have already survived to the specified index age. Confidence intervals (95% CI) are provided where available. Due to limited follow-up, upper confidence intervals for UIA patients at ages 70 and 75 were not fully estimable (NA), and median survival beyond age 80 could not be reliably determined.

Index age (years)	Cohort	Median life expectancy (years)	95% CI
50	Treated UIA cohort	73.9	69.9–79.1
	Matched population	87.2	86.6–87.7
55	Treated UIA cohort	76.7	73.2–81.9
	Matched population	87.2	86.7–87.9
60	Treated UIA cohort	76.7	73.2–81.9
	Matched population	87.4	87.0–88.0
65	Treated UIA cohort	77.1	76.0–85.8
	Matched population	87.7	87.2–88.2
70	Treated UIA cohort	81.8	77.1–NA
	Matched population	88.1	87.6–88.5
75	Treated UIA cohort	82.2	81.8–NA
	Matched population	88.5	88.1–89.3

### 3.2 Causes of death

**Fig. 3:**
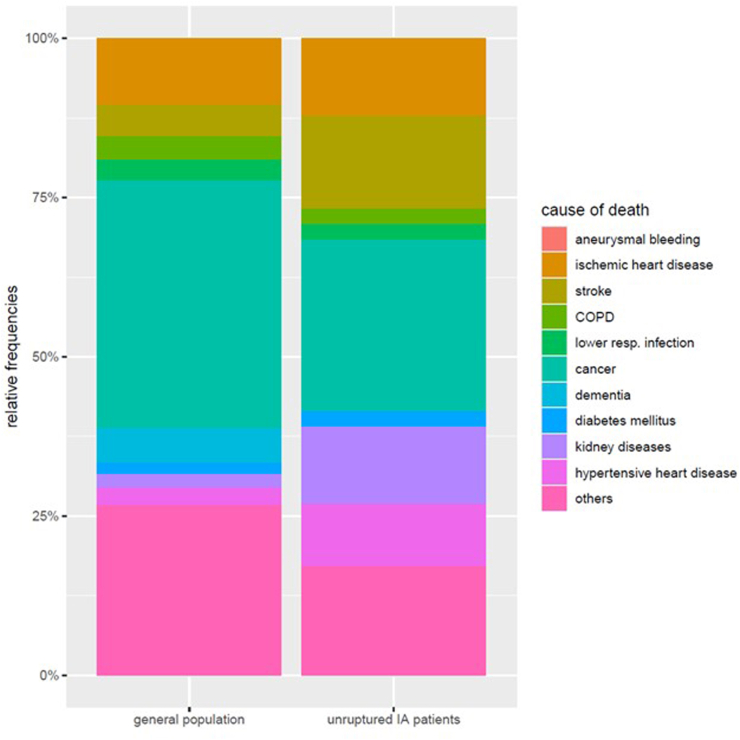
Relative frequency of causes of death among UIA patients and the matched general population, categorized by the most common causes of death in high-income countries, as defined by the World Health Organization (WHO).

Of the 51 deaths among patients with UIAs in the full dataset, n = 42 occurred between the end of 2010 and the end of 2020 and could be matched with the general population. In 7 cases, the cause of death remained unclear. Accordingly, the study on cause of death was performed on n = 35 UIA patients and 4305 matched individuals from the general population. The mean common follow-up time for UIA patients and matched population was 4.5 years (with a median value at 4.1 years). In the matched cohort, the most frequent cause of death was cancer (36.5%), which, although considerably lower, was still the most frequent single cause of death in patients with UIAs (28.6%) (Fig. 3). In contrast, cardiovascular deaths (ischemic heart disease, stroke, diabetes mellitus, and hypertensive heart disease) were more common in UIAs (34.3%) than in the matched population (21.3%). Absolute frequencies for all causes of death are given in Supplementary Table 1.

### 3.3 Prevalence of cardiovascular risk factors among UIA patients

Nearly 60% of patients with UIAs suffered from hypertension. Likewise, hypercholesterinemia (52.4%), active tobacco abuse (45.3%), diabetes mellitus (11.5%), hypercholesterinemia (52.4%), and obesity (38.2%) were more common among patients with UIAs than in the general population (p < 0.001). Table 3 provides an overview of the presence of cardiovascular risk factors and for UIA patients and the general population.

**Table 3:** Presence of cardiovascular risk factors in patients with UIAs compared to the general population Cardiovascular risk factors and co-morbidities at first diagnosis. Presence of these risk factors in the general population are extracted from the Swiss Health Survey 2022. Categorical data are given as absolute frequencies (%). The differences between groups were analyzed by chi-square test. Whereas most cardiovascular risk factors were significantly more prominent in the UIA patients than in the general population, regular alcohol consumption was more prevalent in the general population.

		UIA patients	General Population	p-value
n		382	1190409	
Arterial hypertension (%)		224 (58.6)	463069 (38.9)	<0.001
Smoking				<0.001
	History of smoking (%)	64 (16.8)	333315 (28.0)	
	Active smoking (%)	173 (45.3)	296412 (24.9)	
	Never smoked (%)	145 (38.0)	560683 (47.1)	
Diabetes mellitus (%)		44 (11.5)	79757 (6.7)	<0.001
Hypercholesterinemia (%)		200 (52.4)	232130 (19.5)	<0.001
Obesity (BMI >30) (%)		146 (38.2)	173800 (14.6)	<0.001
Regular alcohol consumption (at least 1x/week) (%)		90 (23.6)	727340 (61.1)	<0.001
Any form of cardiovascular disease (%)		113 (29.6)	328553 (27.6)	0.419

## 4 Discussion

While excess mortality after aneurysmal rupture is well established (Nieuwkamp et al., 2011, 2014; Huhtakangas et al., 2015; Fernandez-Perez et al., 2022), our findings indicate that patients with UIAs also experience reduced long-term survival compared with the general population. During follow-up, UIA patients had a more than threefold higher mortality than age- and sex-matched controls. On average, death occurred 13 years earlier (median 73.9 years) than in the matched population (87.0 years). Cardiovascular comorbidities were common among UIA patients and cardiovascular disease accounted for a substantial proportion of deaths, underscoring the relevance of long-term vascular health in this population.

### 4.1 Long-term mortality in patients with UIAs

In our cohort, overall mortality during follow-up was 13%, with cardiovascular disease accounting for approximately one third of deaths. Data on mid- and long-term survival in patients treated for UIAs remain limited, but previous studies provide important context. A Finnish cohort of 142 untreated IA patients diagnosed between 1956 and 1978 reported a 36% mortality over 13 years, with half of all deaths attributed to cardiovascular causes (Juvela and Lehto, 2015). Advanced age, male sex, smoking, and heavy alcohol consumption were identified as contributors to mortality, with cardiovascular disease emerging as the dominant cause, particularly among men. Another Finnish study including 140 patients diagnosed between 1989 and 1999 and managed conservatively, surgically, or endovascularly observed a 70% excess of cerebrovascular and cardiovascular deaths among deceased patients (mean age 54 years) (Pyysalo et al., 2013). Differences between these historical cohorts and our findings are likely multifactorial. Over recent decades, substantial improvements in lifestyle awareness and cardiovascular disease management have occurred (Sorrentino et al., 2022).] Nevertheless, cardiovascular comorbidities remain frequent in patients with UIAs, suggesting that an aneurysm often coexists with more widespread vascular pathology rather than acting as an isolated determinant of mortality. In this context, detection of a UIA may be viewed as an indicator of broader vascular disease affecting multiple organ systems. In addition, incidental detection of UIAs has increased markedly with wider use of neuroimaging, and treatment indications have evolved considerably over time. For example, prior to 1979, UIAs in Finland were managed exclusively conservatively. Consequently, earlier studies differ substantially from contemporary cohorts with respect to patient selection, treatment strategies, and comorbidity profiles. Despite these differences, the overall direction of the evidence consistently points toward reduced long-term survival in patients with UIAs.

### 4.2 Cardiovascular outcomes in the era of improved procedural safety

The management of UIAs has changed substantially since publication of the ISUIA study (International Study of Unruptured and Intracranial Aneurysms I, 1998), which identified age as the sole independent predictor of poor surgical outcome and encouraged more conservative treatment approaches in older patients. Notably, the mean age at diagnosis in the ISUIA cohort was relatively young at 52 years. Subsequent risk stratification tools, such as the PHASES score (Greving et al., 2014), were derived from populations that differ considerably from today's UIA patients in Western countries. More recently, advances in microsurgical and endovascular techniques - including improved devices, indocyanine green videoangiography, intraoperative digital subtraction angiography, and neuromonitoring - have substantially reduced periprocedural morbidity and mortality and expanded treatment eligibility, even among elderly and multimorbid patients. While these developments have clearly improved short-term safety, their impact on mid- and long-term cardiovascular outcomes remains uncertain. This highlights the importance of continued evaluation of long-term survival and systemic health in patients treated for UIAs. In our cohort, a relatively high proportion of treated aneurysms were located along the ICA, despite the generally lower rupture risk associated with many ICA aneurysms compared with anterior or posterior communicating artery locations. This likely reflects contemporary individualized treatment paradigms, in which treatment decisions are influenced not solely by rupture risk estimates, but also by aneurysm morphology, interval growth, patient-specific vascular risk factors, family history, procedural feasibility, and patient preference. In addition, the increasing incidental detection of ICA aneurysms through widespread use of high-resolution neuroimaging may contribute to their overrepresentation in treated modern cohorts. Therefore, aneurysm location alone should not be interpreted as the sole determinant of treatment indication, and the aneurysm distribution observed in this study likely reflects broader preventive treatment strategies in contemporary neurovascular practice.

### 4.3 Procedure-related complications and long-term survival

Procedure-related complications are an important consideration in UIA management, as they may theoretically affect long-term survival. Earlier reports from the ISUIA study described 1-year morbidity and mortality rates of 10.1% and 2.7% for surgical clipping, and 8.3% and 1.5% for endovascular coiling, respectively, in patients without prior subarachnoid hemorrhage (Wiebers et al., 2003). Since then, outcomes have improved substantially. A meta-analysis by Naggara et al. including approximately 5000 patients reported pooled morbidity and mortality rates of 4.8% and 1.7% following endovascular treatment (Naggara et al., 2010). Similarly, Brinjikji et al. reported procedure-related morbidity and mortality rates of 5% and 4% in patients treated with flow diverters, predominantly for unruptured aneurysms (Brinjikji et al., 2013). Large database analyses and contemporary single-centre series have demonstrated even lower mortality rates, with in-hospital mortality as low as 0.2% following microsurgical treatment (Barker et al., 2003; Catapano et al., 2023). In our cohort, 90-day mortality after UIA treatment was 0.3%, and the single death observed was attributable to advanced comorbid disease rather than the procedure itself. The demonstrated low periprocedural mortality is consistent with modern advances in microsurgical and endovascular treatment. Although comprehensive long-term morbidity data were not uniformly available for all patients, contemporary procedural complication rates alone are unlikely to explain the marked excess mortality observed over prolonged follow-up. Rather, the pronounced burden of systemic cardiovascular comorbidity appears to represent the more substantial determinant of reduced long-term survival in this population.

### 4.4 Decision-making based on quality-adjusted life expectancy

Treatment decisions for asymptomatic patients with incidentally detected aneurysms can be guided by different conceptual frameworks. Increasingly, emphasis has shifted toward outcome-based approaches that consider quality-adjusted life years (QALYs), rather than solely comparing procedural risks with annual rupture probabilities (Raabe et al., 2024). This perspective recognizes that the benefits of preventive UIA treatment may accrue gradually over time and may be influenced by competing health risks. As such, the potential benefit of preventive intervention may be smaller than suggested by traditional survival-based models, particularly in patients with limited life expectancy due to comorbid conditions.

### 4.5 Strength and limitations

This study is based on a robust dataset with a substantial number of patients, minimal loss to follow-up, and high-quality 1:100 matching with the general population. The extended follow-up period of 16 years inevitably encompassed changes in exposure and management of cardiovascular comorbidities, but these are unlikely to have affected the primary analyses of survival and causes of death. Sex-specific analyses were not performed to avoid increased uncertainty, particularly among men, and because age at diagnosis has a stronger influence on remaining life expectancy. Furthermore, while UIA patients appear to represent a population with increased cardiovascular burden, the extent to which excess mortality can be directly attributed to the aneurysm itself remains uncertain. More granular comorbidity matching would be informative but was not feasible due to limitations in population-level data availability. Likewise, detailed retrospective assessment of permanent treatment-related morbidity or subtle functional decline was not consistently feasible across the entire cohort and therefore could not be directly incorporated into survival analyses. As a result, the potential contribution of procedure-related morbidity to long-term survival cannot be fully excluded. Finally, Switzerland ranks among the countries with the highest life expectancy worldwide, reflecting favorable socioeconomic conditions and broad healthcare access. Consequently, the absolute life expectancy estimates observed in our cohort and matched controls may not directly generalize to populations with lower baseline survival, particularly in lower-income countries or healthcare systems with differing preventive vascular care. In countries with shorter baseline life expectancy, the absolute reduction in years of survival associated with UIAs may differ quantitatively. However, the markedly increased relative mortality observed in our cohort, together with the overrepresentation of cardiovascular comorbidities, likely reflects broader systemic vascular vulnerability that may remain relevant across developed healthcare systems. Nevertheless, international validation in more diverse populations is warranted before extrapolating these findings globally.

## 5 Conclusion

Overall, patients with a treated UIA harbor a substantially increased risk of death, and they die, on average, 13 years younger than the general population. Furthermore, as cardiovascular risk factors and cardiovascular deaths are over-proportionally represented among IA patients, aggressive management of all cardiovascular comorbidities appears to be of utmost importance in this patient group.

## Data availability statement

All data supporting the findings of this study are available within the paper and its Supplementary Information. Additional raw data are available from the authors upon reasonable request.

## Author's contribution

Conceptualisation: BEG, SM, HJS; data curation: BEG, BB, SW, PG, CB; formal analysis: BEG, BB, MC, JB, LA; methodology: BEG, SM, LA, HJS; MC; project administration: BEG, LA; resources: GAS, LRem; software: MC; supervision: GAS LReg, LRem, SM, LA, HJS; validation: JB, LA, HJS; visualization: MC, BEG; writing – original draft: BEG; writing – review & editing: BB, SW, PG, JB CB, MC, GAS LReg, LRem, SM, LA, HJS.

## Declaration of generative AI and AI-assisted technologies in the manuscript preparation process

ChatGPT version 5 was used solely for final proofreading of grammar and syntax. No content was generated or drafted by the AI.

## Funding

This research did not receive any specific grant from funding agencies in the public, commercial, or not-for-profit sectors.

## Declaration of competing interest

The authors declare that they have no known competing financial interests or personal relationships that could have appeared to influence the work reported in this paper.
