# Supplementary material for: Long-term survival after treatment of unruptured intracranial aneurysms: A population-based perspective
Source: Brain Spine. 2026 May 19;6:106096. doi: 10.1016/j.bas.2026.106096 (PMC13199678; doi:10.1016/j.bas.2026.106096)

**Long-Term Survival After Treatment of Unruptured Intracranial Aneurysms: A Population-Based Perspective**

**Supplemental Data**

**Table of content:**

- Supplementary Table 1: Cause of Death between end of 2010 and end of 2020 for IA patients and matched population.
- Supplementary Figure 1: life expectancy for IA patients and matched cohort.

**Supplementary Table 1: Cause of Death for UIA patients and matched population.**

Absolute frequencies (%).

|  | **UIAs** | **matched** |
| --- | --- | --- |
| n | 35 | 1526 |
| cause of death |  |  |
| aneurysmal bleeding | 0 (0.0) | 0 (0.0) |
| ischemic heart disease | 4 (11.4) | 160 (10.5) |
| stroke | 4 (11.4) | 76 (5.0) |
| COPD | 1 (2.9) | 55 (3.6) |
| lower respiratory infection | 1 (2.9) | 49 (3.2) |
| cancer | 10 (28.6) | 601 (39.4) |
| dementia | 0 (0.0) | 84 (5.5) |
| diabetes mellitus | 1 (2.9) | 26 (1.7) |
| kidney diseases | 4 (11.4) | 33 (2.2) |
| hypertensive heart disease | 3 (8.6) | 42 (2.8) |
| others | 7 (20.0) | 400 (26.2) |

**Supplementary Figure 1:** Kaplan–Meier curves of the cumulative relative frequency of deaths for UIA patients (blue) and matched general population (green) with respect to absolute calendar time (years given on the lower x-axis). The hazard of death is significantly higher for unruptured IA patients (blue) than for the general population (green) (p<0.001). A slight increase in overall mortality after 2020 probably reflects excess mortality due to the COVID pandemic.


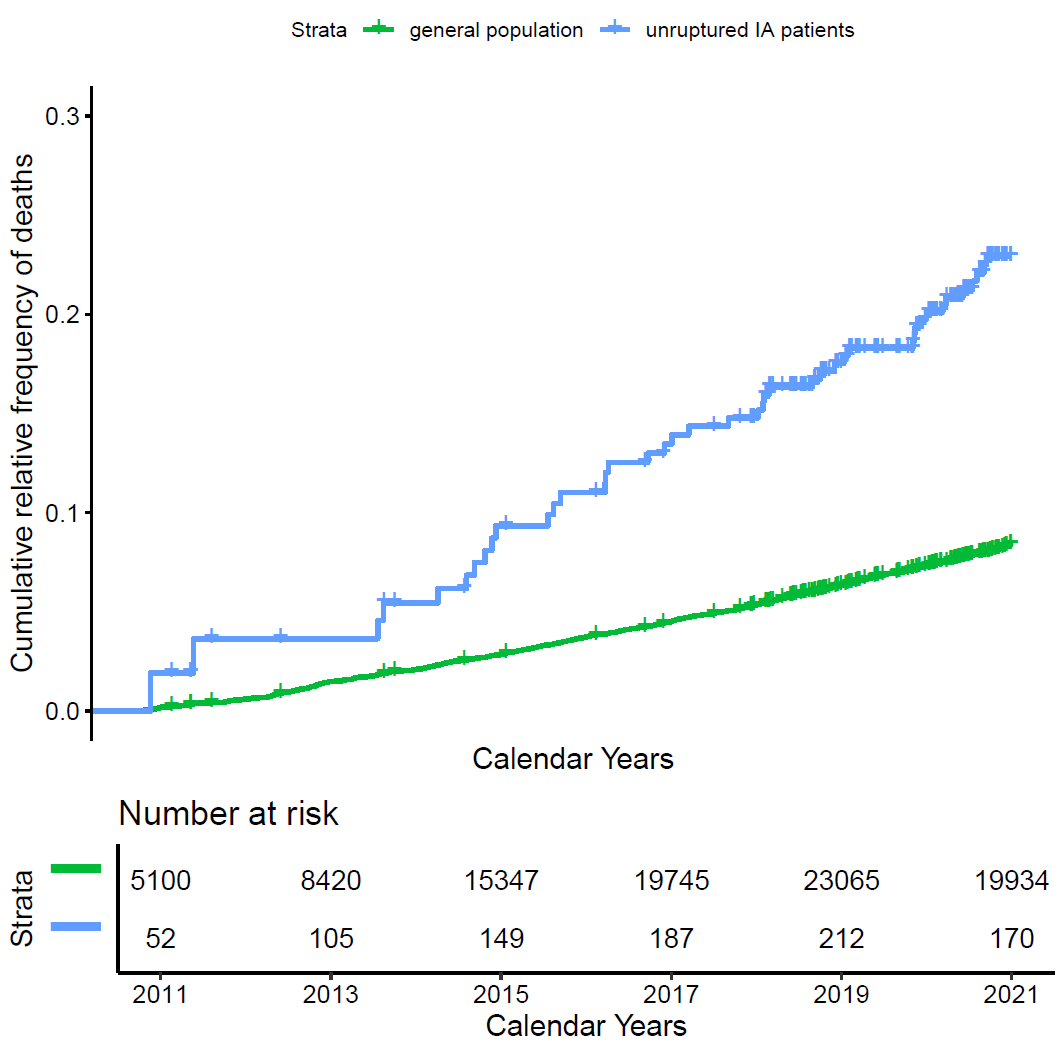

Supplement: Multimedia component 1 [file mmc1.docx]
